# Simple curettage and allogeneic cancellous bone chip impaction grafting in solitary enchondroma of the short tubular bones of the hand

**DOI:** 10.1038/s41598-023-29130-w

**Published:** 2023-02-06

**Authors:** Ho Youn Park, Min Wook Joo, Youn-Ho Choi, Yang-Guk Chung, Chan Jin Park

**Affiliations:** 1 Department of Orthopedic Surgery, Uijeongbu St. Mary’s Hospital, College of Medicine, The Catholic University of Korea, Seoul, Republic of Korea; 2 Department of Orthopedic Surgery, St. Vincent’s Hospital, College of Medicine, The Catholic University of Korea, 93, Jungbu-Daero, Paldal-Gu, Suwon-Si, Gyeonggi-Do 16247 Republic of Korea; 3 Department of Orthopedic Surgery, Seoul St. Mary’s Hospital, College of Medicine, The Catholic University of Korea, Seoul, Republic of Korea

**Keywords:** Medical research, Oncology, Engineering, Materials science

## Abstract

Enchondroma is the most common bone tumor in the hand. While standard surgical procedure is intra-lesional excision and bone grafting, there is a dispute between allogeneic bone, autogenous bone, and synthetic bone substitute grafting. Diverse adjuvant treatments have been introduced to reduce recurrence, but results are mixed with controversies. Meanwhile, whether existing descriptive classification could predict treatment outcome remains unclear. Thus, we reviewed patients with solitary enchondroma of the hand who underwent simple curettage followed by allogeneic cancellous bone chip impaction grafting. Eighty-eight patients with more than 5 years of follow-up were enrolled. Demographic data, local recurrence, and complications were reviewed. Duration of consolidation and the difference according to Takigawa classification were assessed. Range of motion (ROM), and functional scores were also evaluated. There were 51 women and 37 men, with a mean age of 37.9 years. Mean follow-up was 10.2 years. Recurrence occurred only in one patient. There was no complication. Mean postoperative total active motions of fingers and thumb were 239° and 132.9°. Mean modified Disabilities of the Arm, Shoulder, Hand score, and Musculoskeletal Tumor Society Score were 1.63, and 99.2 at the last follow-up. Consolidation, ROM, and functional scores according to Takigawa classification showed no significant differences. This study suggests that simple curettage with impaction grafting of allogeneic cancellous bone chip is a feasible method for treating solitary enchondromas involving short tubular bone of the hand with good long-term outcomes. Postoperative recurrence and complication rates were very low. Radiographic and clinical results were good regardless of the previous radiological classification.

## Introduction

Enchondroma is the most common benign bone tumor in the hand, with 40–47% of cases involving the hand^1–4^. Due to its slow growth, most cases are asymptomatic and incidentally detected. Symptomatic cases are usually in the setting of large lesions or pathologic fractures. Surgery is considered for both histopathologic confirmation and treatment or prevention of pathological fractures^5^. The standard surgical procedure most commonly used is intra-lesional excision combined with bone grafting^6,7^. Bone grafting is performed to promote bone regeneration and restore bone strength for skeletal defects following tumor excision^4,8^.

There is still a dispute between allogeneic bone, autogenous bone, and synthetic bone substitutes grafting. Advocates for allogeneic bone grafting assert that this surgical procedure is relatively straightforward with outcomes comparable to those of autogenous bone grafting^9^. However, there are still concerns about immune response, disease transmission, postoperative infection, and price associated with allogeneic bone grafting. Autogenous bone grafting has demonstrated excellent bone formation without these aforementioned complications. However, donor-site complications might occur^7,10^. Recently, good results of synthetic bone substitutes grafting have been reported^11–14^, but there are still concerns about foreign body reaction, long-term prognosis, and price. Therefore, which surgical method to use may depend on the surgeon’s preference.

Incidence of recurrence following resection and bone grafting is 2–15%^4,15,16^, which is similar between autogenous and allogeneic bone grafting^16^. While diverse adjuvant treatment methods in addition to curettage have been introduced to reduce recurrence, results are mixed with controversies. Therefore, such adjuvant treatments might not be helpful for treating solitary enchondroma of the hand.

In 1971, Takigawa first classified 110 cases of chondroma according to their shapes. Since then, the Takigawa classification has been often used in the literature. It divides chondromas into two types (monostotic and polycentric types) and five forms (central, eccentric, combined, polycentric and giant forms) based on simple radiographic findings^17^. In the literature, this system is mainly used for morphological classification. However, whether the descriptive classification could predict treatment outcome or prognosis remains unclear.

In this study, we aimed to address the following questions pertaining to the solitary enchondroma of the hand: (1) Can simple curettage without any adjuvant treatment prevent long-term recurrence? (2) Is allogeneic cancellous bone chip impaction grafting sufficient to promote bone regeneration with fewer complications? (3) What are clinical implications of conventional radiographic classification methods for solitary enchondroma of the hand?

## Results

### General patient information

Among 88 patients who were enrolled in this study, there were 51 women and 37 men. Their mean age was 37.9 years (range, 13–72 years). All lesions were confirmed as enchondroma by histologic examination. Symptoms included only pain, only deformity, and painful deformity and nineteen patients were diagnosed incidentally. The average duration of symptom before surgery was 3.3 months (range, 1 week–5 months). The mean amount of allogeneic cancellous bone used for surgery was 3 cc (range, 1.5–15 cc). The mean follow-up period after surgery was 10.2 years (range, 5.0–15.6 years). In the follow-up period, recurrence occurred only in one patient. This patient was a 16-year-old male with a lesion of 16.5 mm and type 1 in the proximal phalanx. A new cystic lesion was found at 26 months after the first operation after he reported recurred pain. Repeated curettage and allogeneic cancellous bone chip impaction grafting were performed. Pathology confirmed the enchondroma.

### Radiographic results

**Figure 1:**
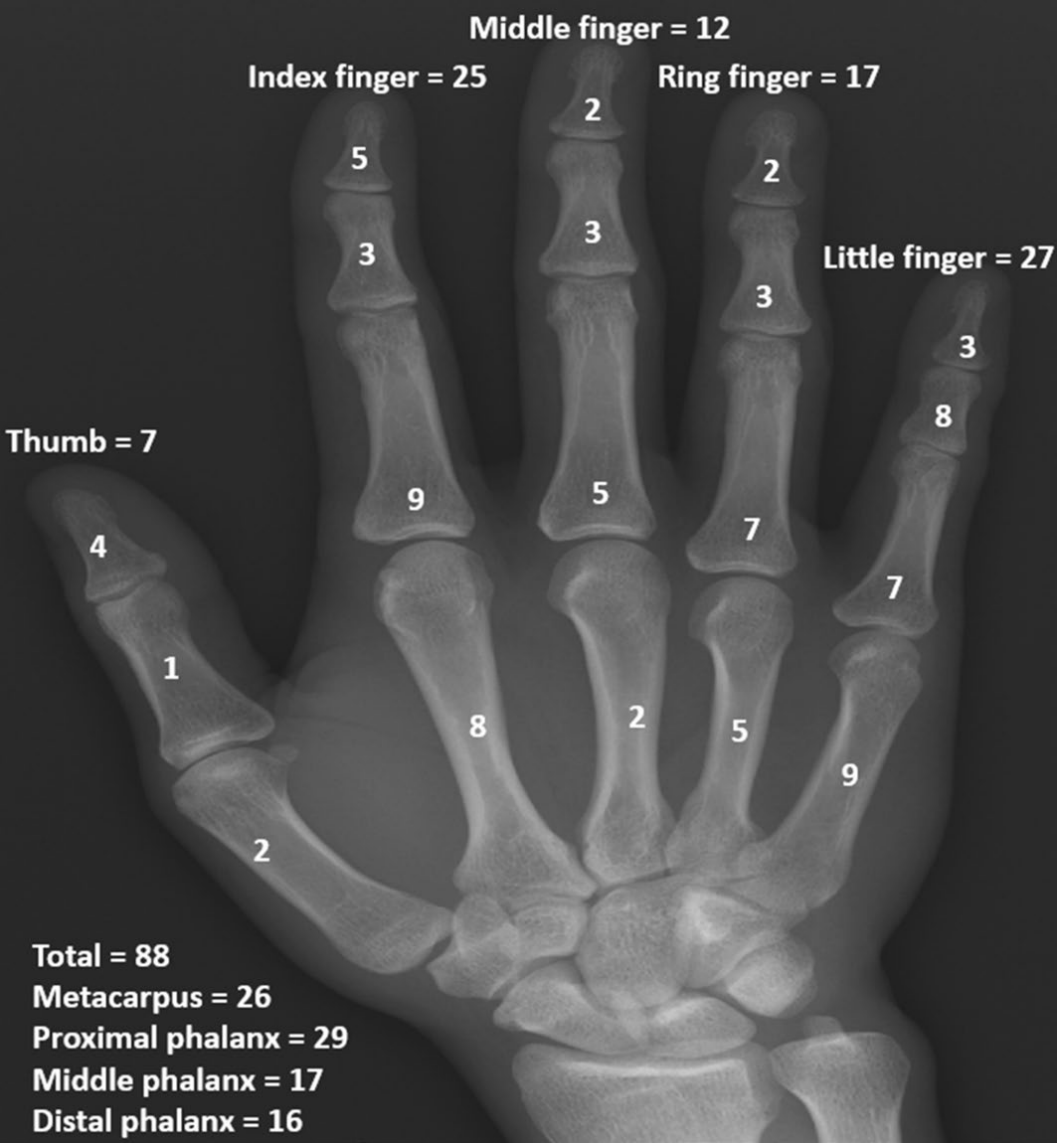
This figure shows locations of lesions. The most common affected sites among the fingers were the index and little fingers. Proximal phalanges were most frequently affected, followed by the metacarpal bones, middle phalanges, and distal phalanges among the bones.

**Figure 2:**
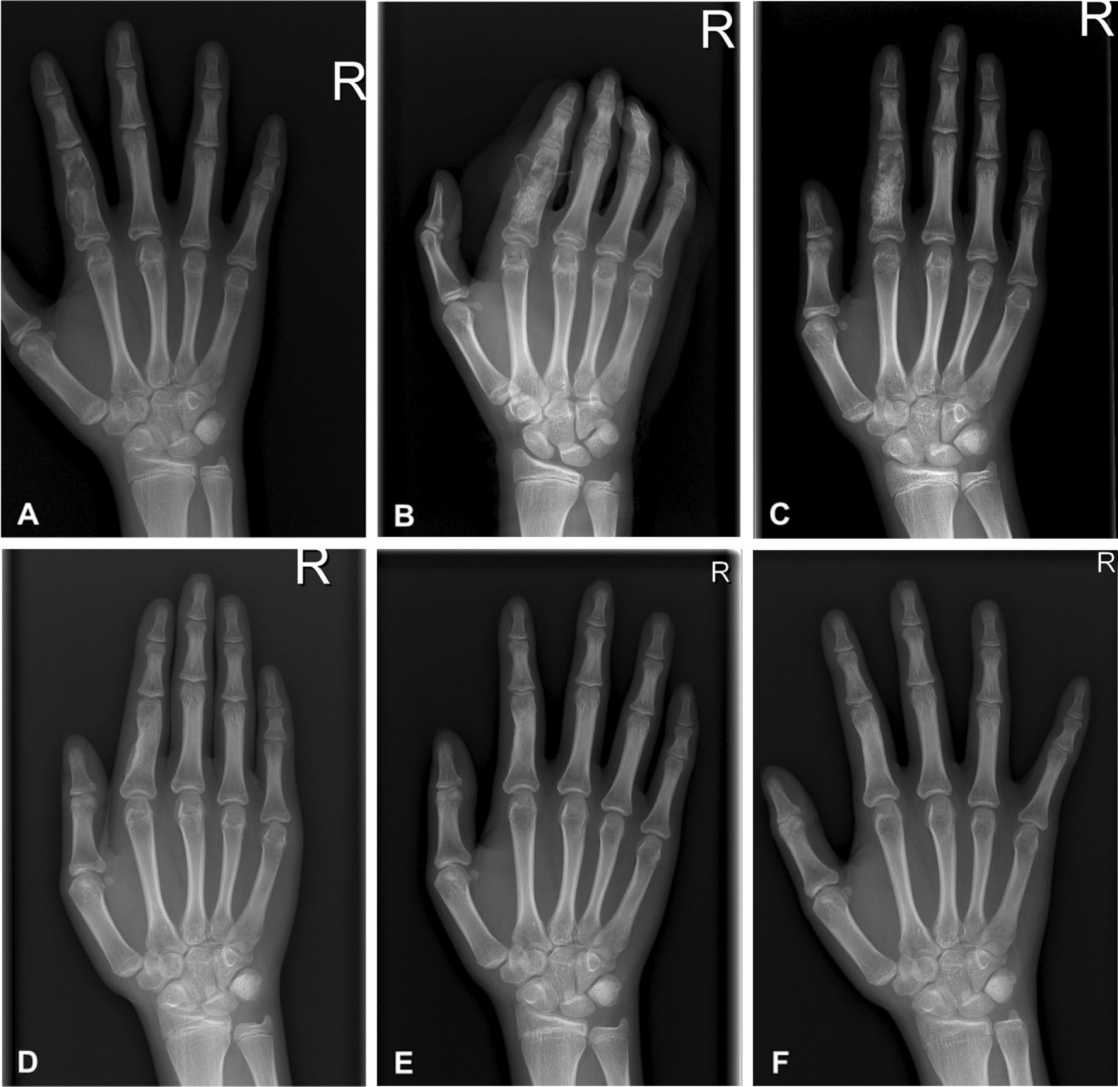
A 13-year-old female with enchondroma of polycentric and giant form in the proximal phalanx of the right index finger. Simple radiographs taken before surgery (**A**), immediately (**B**), 1 month (**C**), 2 months (**D**), 1 year (**E**), and 9 years (**F**) after surgery.

**Figure 3:**
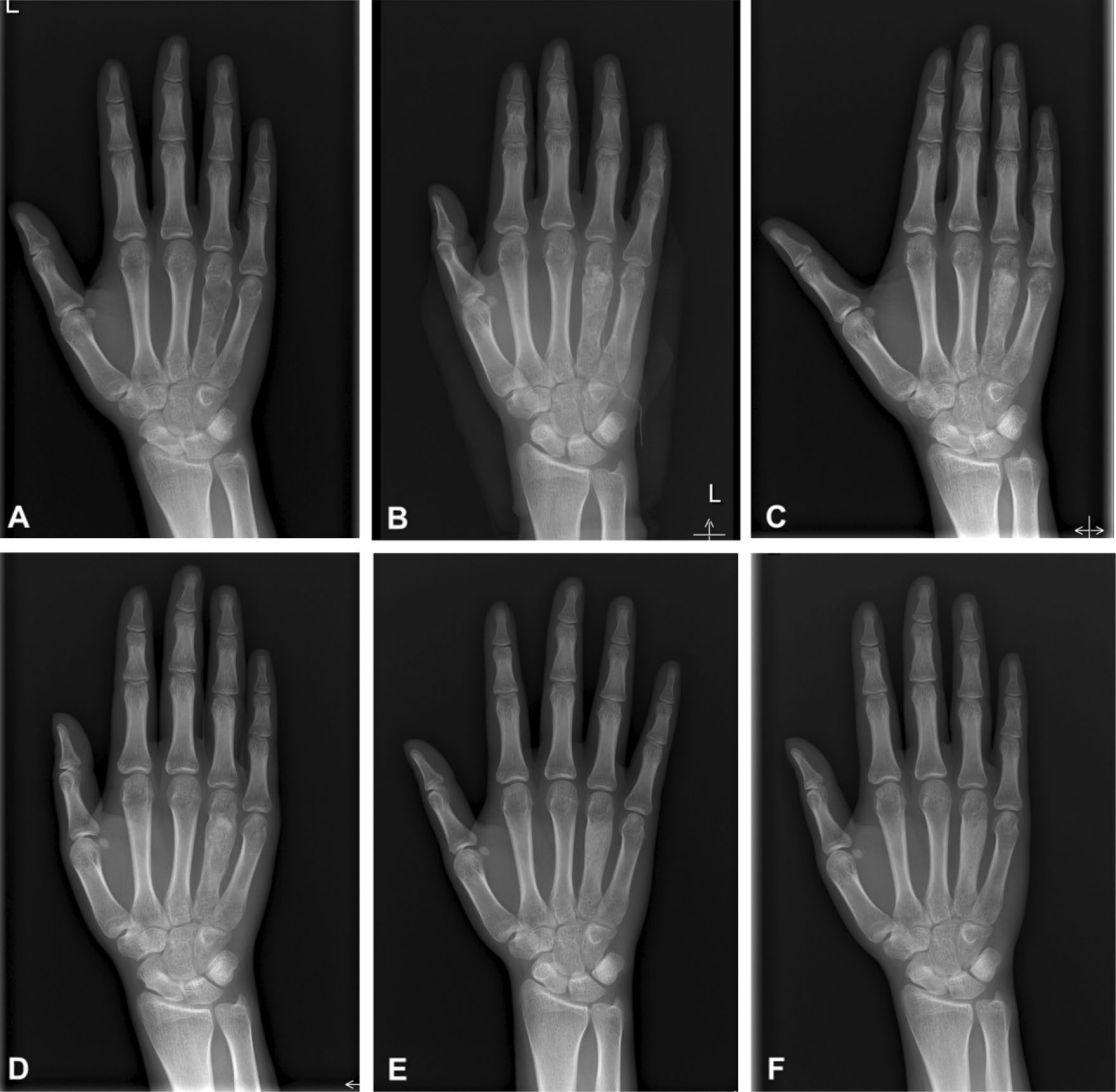
A 18-year-old male with enchondroma of giant form in the fourth metacarpus of the Lt. hand. Simple radiographs taken before surgery (**A**), immediately (**B**), 1 month (**C**), 2 months (**D**), 1 year (**E**), and 6 years (**F**) after surgery.

Proximal phalanges were most frequently affected, followed by metacarpal bones, mid-phalanges, and distal phalanges. The most common affected sites among the fingers were the index finger and the little finger (Fig 1). Mean long axis of the lesion was 15 mm (range, 6–35 mm). The mean radiological consolidation period was 6 weeks (range, 4–12 weeks). There were 23 (26.1%) patients with Takigawa type A, 35 (39.8%) patients with type B, 21 (23.9%) patients with type C, and 9 (10.2%) patients with type D. The difference in time for radiological consolidation according to the Takigawa classification was not significant (*p* = 0.166) (Table 1) (Figs 2 and 3).

**Table 1:** Radiological and clinical outcomes according to Takigawa classification.

Classification	Consolidation period (months)	ROM* (°)	Modified DASH score*	MSTS score*
Type A (n = 23)	4.0 (3–12)	240.7 (210–250)	1.6 (0–6.8)	98.8 (93.3–100)
Type B (n = 35)	3.17 (3–12)	239.7 (210–250)	2.6 (0–6.8)	98.6 (90–100)
Type C (n = 21)	3.7 (3–12)	238.7 (210–250)	2.0 (0–4.5)	99.8 (96.7–100)
Type D (n = 9)	4.1 (4–12)	238.3 (220–250)	2.3 (0–9.1)	99.6 (96.7–100)
*p* Value	0.166	0.131	0.206	0.827

*ROM* range of motion of the finger; *DASH* Disabilities of the Arm, Shoulder, and Hand; *MSTS* Musculoskeletal Tumor Society. *Assessments were performed at postoperative 6 months.

### Functional results

Postoperative range of motion (ROM) measurements at 6 months after surgery were reported in 85 out of 88 patients. Mean preoperative and postoperative total active motion (TAM) of fingers were 197.9° (range, 80°–250°) and 239.0° (range, 210°–250°), respectively. Mean preoperative and postoperative TAMs of thumb were 124.3° (range, 100°–140°) and 132.9° (range, 120°–140°), respectively. ROMs of fingers according to the Takigawa classification showed no significant difference (*p* = 0.131) (Table 1). Mean preoperative modified Disabilities of the Arm, Shoulder, and Hand (DASH) score and Musculoskeletal Tumor Society (MSTS) score were 2.07 (range, 1–9.1) and 84.7 (30–100), respectively. Mean modified DASH score and MSTS score at the last follow-up visit were 1.63 (range, 0–5), and 99.2 (range, 90.0–100), respectively. These two scores at the last follow-up according to the Takigawa classification showed no significant difference (*p* = 0.206, and *p* = 0.827) (Table 1).

### Complications

After surgery, there was no complication associated with allogeneic bone grafting such as immune response, disease transmission, or postoperative infection until the final follow-up.

## Discussion

There is debate about which surgical method is superior for treating solitary enchondroma of the hand. Whether curettage alone is sufficient and whether allogeneic or autogenous bone grafting is the optimal approach remains unclear. The classification suggested by Takigawa^17^ is commonly used for morphological descriptions. However, whether this classification system can predict treatment outcomes has not been demonstrated in large-scale case series. Our study analyzed long-term (> 5 years) clinical outcomes following simple curettage and allogeneic cancellous bone chip impaction grafting in 88 patients with a solitary enchondroma of the hand. In our experience, there was a good bone formation with only one documented recurrence. There were no complications associated with allogeneic cancellous bone chips. Our results also revealed no significant differences in duration of consolidation, ROM, or postoperative clinical scores according to the Takigawa classification.

Adjuvant treatments such as high-speed burring, alcohol soaking, CO_2_ laser ablation and bone cementation can be used to reduce risk of recurrence after curettage for endochondroma of the hand^4,17–19^. A previous study has reported that the difference in recurrence is not significant between case with alcohol treatment and those with high-speed burring^20^. Another research has revealed no treatment-related complications and recurrence when additional therapy with CO_2_ laser is performed after curettage^19^. In this study, recurrence developed only in one out of 88 patients even without adjuvant treatment after curettage using curettes. Considering surgical time and effort required for additional therapy, simple curettage is regarded a more efficient operational procedure than curettage followed by additional treatment.

While autogenous bone transplantation is still used to reconstruct a bone defect after curettage in the hand enchondroma, it might have donor site problems such as infection, hematoma, pain, and paresthesia^10^. In addition, operative time is prolonged^10^. Anterior iliac crest bone is preferred for autografting in enchondroma of the hand. Complications have been much reduced with the development of surgical instruments and techniques, but donor site morbidity was still reported as high as 7%^21^ after anterior iliac crest bone harvesting. It was reported that three patients complained of scar numbness among 37 with an average follow-up of 4.5 years^22^. In children who underwent iliac bone harvesting, school was resumed after an average of 12.6 days and sport activities after 1 month due to pain regardless of the used pain management protocol^23^. While bone can be harvested from the distal radius, there may still be donor site morbidity and harvest volume can be limited^24^. A study comparing curettage alone, autografting, bone substitute insertion, and cementation reported that the complication rate was 0.7, 3.5, 0, and 2.0%, respectively^25^. There is no significant difference in recurrence, but complications such as infection and donor site pain were prominent in case of autografting^12^. In that sense, the use of allogeneic bone or synthetic bone substitute grafting is increasing. Widely used synthetic bone substitutes are based on calcium phosphate, calcium sulphate, and the products are available in various forms such as granular and injectable ones^12,25^. They are convenient to use, however, possible disadvantages are foreign body reaction, delayed integration, insufficient defect consolidation, and lack of mechanical strength^26^. It was reported that one case of foreign body reaction among 11 patients with enchondroma who had synthetic bone substitute grafting^12^. Although allogeneic bone might potentially lead to immunological problems and disease morbidity, currently no such cases have been reported in previous studies^7,27^. Such complications did not occur in the present study, either.

As for consolidation period, autogenous bone grafting is the fastest, but allogeneic bone grafting is comparable. A study reported that the autografts took a mean of 38 days and allograft took a mean of 51 days for consolidation in 76 patients with 15-year follow-up period^8^. In the current study, the mean radiological consolidation period was 6 weeks (range, 4–12 weeks). There are still controversies on the timing of bone integration, and it can take 9 ~ 12 months in synthetic bone grafting^12^. While it took only 4 weeks to achieve radiologic consolidation in a case report^11^, but the outcome is difficult to be reliable due to the small number of cases.

Meanwhile, we should consider that early movement is important to prevent finger contracture in the treatment of hand enchondroma. Thinner cortical bone can increase the risk of fracture and prolong the immobilization period. Since contractures can occur with prolonged immobilization, it is necessary to have some stability after curettage. Therefore, augmentation after curettage might be needed. Bone cement such as PMMA, hydroxyapatite, Plaster of Paris can be used to fill the cavity for augmentation^4,28,29^. PMMA provides immediate mechanical strength to withstand weight bearing^30^. Cement injection after curettage has the advantage of enabling early joint motion. However, since the hand primarily does not bear a body weight, such immediate high mechanical strength is not required for finger motion. In addition, the incidence of infection and cellulitis caused by PMMA is higher than that by allogeneic bone^30^. We believe that impaction procedure could provide more structural stability in bone grafting^31^. It can also promote bone regeneration unlike other materials. Therefore, allogeneic cancellous bone chip impaction grafting is considered to be a safe and more efficient surgical technique for treatment of solitary enchondroma of the hand.

Since the first introduction of a morphological classification for hand enchondroma^17^, several studies have reported on its types and forms. While some reports^7,12,16,17^ showed that the central form was the most common one with the eccentric form being the second most common followed by the giant form which was the rarest, another study^32^ assessing isolated enchondroma of the hand reported that the eccentric form was the most common one. In the current study regarding the solitary enchondroma involving the short tubular bone of the hand, the most common form was the eccentric form, followed by the central form. There was no giant form. While studies on the treatment results according to classification are still lacking, there were no significant differences in consolidation period, ROM, or postoperative clinical scores according to the Takigawa classification in this study.

The current study had several limitations. First, this was a retrospective case series without a control group. We could not compare results of other surgical methods as the authors prefer this surgical method for solitary enchondroma of the hand and rarely use other surgical methods for a long time. In this study, 38 cases using other surgical methods were excluded. Although curettage followed by autogenous bone grafting has been considered the standard of surgical care, major complications might occur at the donor site^33^. To the best of our knowledge, we are the first to report long-term outcomes of allogeneic bone grafting with such a large number of cases. Considering previous reports on autografting, our method does not seem to be inferior as it has no donor site problems^7,9^. The second limitation was its retrospective study design. Therefore, some data were missing. Although the surgeon tried to follow up the patients at regular intervals, the consolidation period might not be accurate because it was evaluated at the time of the patient’s visit. Currently, the authors are conducting a prospective trial comparing autogenous and allogeneic bone grafting. Moreover, 93 patients with a follow-up of less than 5 years were excluded, which might have led to a selection bias. However, it is more common for patients with solitary enchondroma of the hand to fail to follow up when they are well without experiencing postoperative problems. Besides, local recurrence did not occur during their follow-up period in the 93 excluded patients. In this paper, only patients who were followed up for more than 5 years were included, demonstrating that the results of this surgical method were maintained well in the long term.

In conclusion, this study suggests that simple curettage with impaction grafting of allogeneic cancellous bone chip is a feasible surgical method for treating solitary enchondromas involving short tubular bone of the hand with good long-term outcomes. Postoperative recurrence and complication rates were very low. Radiographic and clinical results were good regardless of the radiological classification of lesions. Although previous classification is useful for explaining the lesion, but it may not affect treatment outcomes.

## Methods

### Study design and participants

In this retrospective, single-center study, chart review was performed to identify patients with histologically proven solitary enchondroma involving the short tubular bone of the hand who underwent simple curettage followed by allogeneic cancellous bone chip impaction grafting. The Catholic University of Korea St. Vincent’s Hospital Institutional Review Board approved this study (VC22RASI0161) and waived the need for informed consent as this retrospective medical record review was deemed a minimal risk study without collecting personal identifiable information. The research was carried out according to guidelines and regulations of the ethic committee.

**Figure 4:**
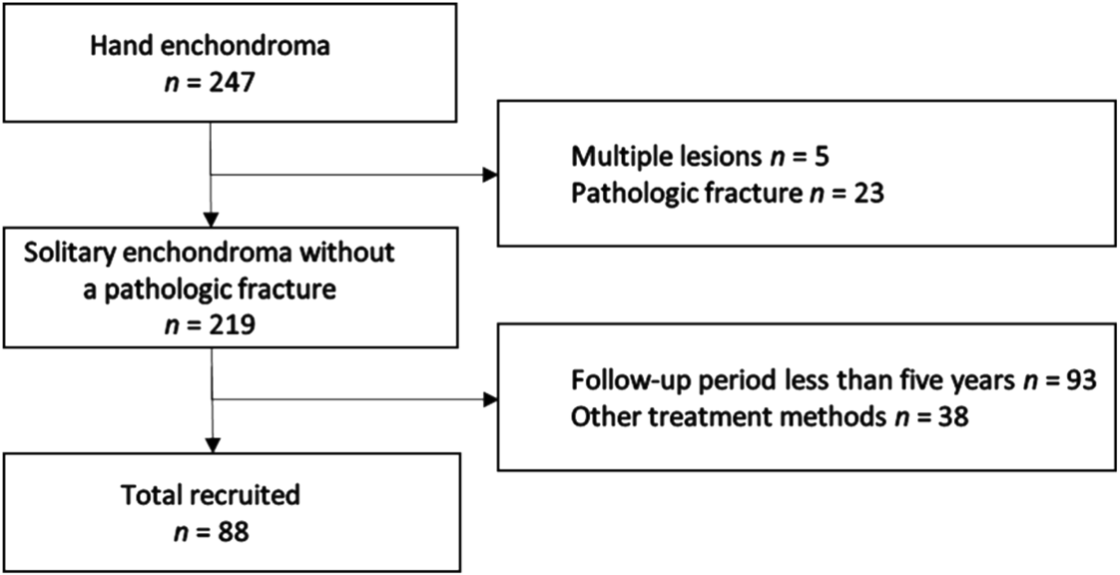
STROBE diagram demonstrates the patient selection process.

Inclusion criteria were patients (1) with histologically proven solitary enchondroma involving the short tubular bone of the hand, (2) without pathologic fractures, (3) who underwent simple curettage and allogeneic cancellous bone chip impaction grafting, and (4) with postoperative follow-up period longer than 5 years. Therefore, patients (1) with multiple lesions, (2) pathologic fractures and (3) follow-up period shorter than 5 years, and (4) who had autogenous bone grafting or any adjuvant treatment were excluded. From January 2006 to December 2016, 247 consecutive patients were diagnosed with enchondroma of the hand. Five patients with multiple enchondromas, and 23 patients with pathologic fractures were excluded. In addition, 93 patients who had insufficient follow-up period (< 5 years) and 38 patients who had autogenous bone grafting or any adjuvant treatment were excluded. Finally, a total of 88 patients who underwent simple curettage followed by allogeneic cancellous bone chip impaction grafting without any adjuvant treatment were enrolled in this study (Fig 4).

### Surgical procedure

Simple curettage followed by allogeneic cancellous bone chip impaction grafting was performed. When the lesion was located on the phalanx, a dorsal or a lateral approach was used. A dorsal approach was used for metacarpal lesions. A large window was made in the cortical bone with a burr under fluoroscopy to confirm the extent of the lesion. Thorough curettage using curettes was performed to completely excise the lesion until cortical bone or normal cancellous bone was visible. The surgical specimen was sent to the hospital pathology department for histologic evaluation. After saline irrigation of the surgical site, allogeneic cancellous bone chip impaction grafting was performed using fluoroscopic guidance. After surgery, the hand was immobilized with short arm splint for approximately 2 weeks.

### Radiographic evaluation

Patients were usually scheduled to have follow-up visits every 4 weeks during the first 3 months. Patients were then instructed to follow up every 3–6 months during the first year on a case-by-case basis. A yearly follow-up visit was then recommended. At each visit, patients underwent radiographic and clinical evaluations. Preoperative anteroposterior (AP), lateral, and oblique plain radiographs of the affected hand and postoperative radiographs obtained during follow-up period were evaluated. Preoperatively, the anatomical location of the lesion, size of the lesion, and Takigawa classification^17^ were assessed. Lesions were categorized into four types: central (type A), eccentric (type B), combined (type C), and polycentric or giant (type D).

Radiographic consolidation was defined by the presence of normal cortical bone and if the bone defect was less than 3 mm on plain radiographs after surgery. This finding corresponds to grade I of Tordai classification^34^ (grade I, normal cortical bone or a bone defect smaller than 3 mm in diameter; grade II, bone defects with a diameter 4 to 10 mm; grade III, bone defect larger than 10 mm with characteristics of enchondroma). We reviewed the duration of consolidation and analyzed the difference in duration according to the Takigawa classification of the lesion. Recurrent lesions were defined if the patient reported new pain or a new cystic change was noted on follow-up radiograph, and an enchondroma was histologically re-confirmed after re-operation.

### Clinical evaluation

Age, sex, and preoperative symptom duration were reviewed. ROM was assessed using TAM of the affected digit. TAM was defined as the sum of active flexion and extension motion of metacarpophalangeal (MCP), proximal interphalangeal (PIP) and distal interphalangeal (DIP) arc of motion in degrees. Normal TAMs of the finger and thumb were reported as 260 and 140 degrees, respectively. We reviewed records of preoperative ROM and ROM at 6 months after surgery, as it was expected that there may be no significant changes in ROM after postoperative 6 months. Modified DASH score^35^ and MSTS score^36^ were evaluated. We analyzed the difference in ROM and the functional scores according to the Takigawa classification. Complications associated with allogeneic bone graft, including immune response, disease transmission, and postoperative infection, were reviewed.

### Statistical analysis

Continuous variables are presented as means and ranges. Categorical variables are presented as numbers and percentages. One-way analysis of variance (ANOVA) was used to analyze the differences in postoperative consolidation period, ROM, modified DASH and MSTS scores according to the Takagawa classification. *P* value less than 0.05 was considered statistically significant.

## Data Availability

All data generated or analyzed during this study are included in this published article.
